# Digital Support for Cancer Caregivers in Latin America: Identifying Needs and Co‐Designing a Culturally Adapted Resource

**DOI:** 10.1002/pon.70609

**Published:** 2026-09-17

**Authors:** Sonia Carreño‐Moreno, Lorena Chaparro‐Diaz, Carolyn Taylor, Olinda Santin

**Affiliations:** ^1^ Facultad de Enfermería Universidad Nacional de Colombia Bogota Colombia; ^2^ Global Focus on Cancer South Salem New York USA; ^3^ School of Nursing and Midwifery Queen's University Belfast Medical Biology Centre Belfast UK

**Keywords:** cancer caregivers, co‐design, digital health, Latin America, low‐ and middle‐income countries, psychosocial support

## Abstract

**Objective:**

Cancer caregivers in low‐ and middle‐income countries face substantial psychosocial and practical burdens. In Latin America, caregivers provide essential support under resource constrained conditions. This study aimed to co‐design a transferable digital resource for Spanish‐speaking caregivers that reflects their support and information needs.

**Methods:**

A three‐phase co‐design study engaged caregivers, healthcare professionals, academics, and policymakers across Latin America including Colombia, Argentina, Mexico and Peru. Phase 1 involved a hybrid workshop with oncology professionals to identify and prioritise caregiver needs. Phase 2 included verification workshops with professionals and an online caregiver survey to refine content and delivery. Phase 3 used think‐aloud workshops to evaluate and finalise the prototype.

**Results:**

A total of 342 stakeholders contributed. Participants highlighted the need for accessible, structured support that addressed evolving caregiving needs. While information, practical support, and healthcare navigation were prioritised, participants also emphasised the importance of peer connection and maintaining opportunities for face‐to‐face engagement, positioning digital resources as a complement rather than a replacement for human support. Stakeholders highlighted the need for inclusive and accessible design, including simplified language, engaging multimedia content, caregiver narratives, and representation of diverse caregiving experiences. The resulting six‐module prototype addressed the caregiver role, practical care and symptom support, navigating health and social care systems, palliative and end‐of‐life care, caregiver well‐being, and locally adaptable supportive resources.

**Conclusions:**

This co‐designed resource provides a foundation for developing culturally responsive caregiver support across Latin America. By combining digital delivery with opportunities for social connection, inclusive design, and local adaptation, the resource addresses key needs while supporting future implementation within existing care systems.

## 1 Introduction

Cancer is a growing public health challenge across Latin America [1]. The Latin America and Caribbean Region (LACR) reports a higher incidence of cancer than many other low‐and middle‐income regions (LMIC's), including Africa and Asia, and experiences mortality rates that substantially exceed those observed in North America [1]. By 2045, both cancer incidence and mortality in the LACR are projected to increase considerably, outpacing forecasts for North America and Oceania [1].

This growing cancer burden is compounded by systemic healthcare challenges. Although countries in Latin America, including Colombia, have achieved universal health insurance coverage, cancer care remains constrained by fragmented health systems, limited service availability, and poor coordination between insurers and providers [2]. Cancer services are also concentrated in major urban centres, contributing to diagnostic delays and poorer outcomes in geographically underserved populations [3]. In addition, limited availability and coverage of population based cancer registries hinder service planning and resource allocation [4]. Consequently, the true cancer burden may be underestimated, obscuring unmet needs and limiting effective health service planning [4].

Within this context, informal caregivers play a critical yet often under recognised role. As cancer prevalence rises across Latin America, family members increasingly provide physical, emotional, practical, and logistical support, often with limited formal assistance, particularly in remote, and socioeconomically disadvantaged communities. In LMICs, deficiencies in resources shift substantial responsibility for cancer care onto caregivers, who frequently undertake symptom monitoring, treatment support, care and coordination with little preparation or support, effectively compensating for gaps in formal services [5, 6]. However, caregivers often lack adequate health literacy and access to psychosocial support, increasing their risk of psychological distress, poor physical and mental health, social isolation, and financial hardship [7, 8]. Importantly, caregiver wellbeing is closely associated with patient outcomes, including treatment adherence, quality of life, and overall care experiences, underscoring the need to address caregivers' unmet needs [9, 10].

Digital interventions offer a promising approach to supporting caregivers in resource constrained settings [11, 12]. They provide scalable, low cost access to support while overcoming workforce and geographical barriers. Digital and structured psychosocial interventions have been shown to improve health literacy, reduce social isolation, and enhance psychosocial well‐being among caregivers [13, 14]. However, caregiver focused digital resources in LMICs remain limited and often lack meaningful end user involvement in their development. Moreover, much of the evidence derives from high‐income countries, limiting its applicability to LMIC cultural and resource contexts [11, 12, 13, 14]. In response, co‐design is an iterative, participatory approach that actively engages end users throughout development is increasingly recognised as essential for ensuring digital interventions are culturally relevant, acceptable, usable, and responsive to local needs in LMIC settings [15, 16, 17].

This study builds on prior co‐designed interventions developed in the UK, Australia, and Vietnam [12, 18, 19]. Our previous work demonstrated that caregivers' needs are shaped not only by language and culture but also by local care pathways and caregiving roles [12, 18, 19]. Given the shared challenges faced by caregivers across Latin America, this study aimed to co‐design a transferable digital resource for Spanish‐speaking caregivers that is responsive to their support and information needs. This paper reports the first three phases of the co‐design process.

## 2 Methodology

### 2.1 Ethical Approval

Ethical approval was obtained from the Research Ethics Committee of the Faculty of Nursing, Universidad Nacional de Colombia (AVAL‐001–25).

### 2.2 Study Design

This study used a co‐design methodology informed by the six‐step model developed by Santin et al. [18]. The model comprises six stages: (1) content generation, (2) prototype development, (3) prototype review, (4) prototype adaptation, (5) usability testing, and (6) final modification. This study focused on the first four stages, culminating in the development and refinement of a written prototype of the digital resource.

Subsequent work will involve digital platform development, usability testing, and final refinement.

#### 2.2.1 Phase 1: Content Generation and Prototype Development (Santin Steps 1–2)

Phase 1 was conceptualised as an expert co‐design workshop with oncology nurses with leading oncology nurses who have responsibility for delivering oncology programmes within their organisations, drawing on their clinical experience and an existing body of research [11, 12, 18, 19, 20, 21] on caregiver needs to generate an initial content framework and implementation considerations. Phases 2‐3 purposefully broadened stakeholder representation to include family caregivers, academics, and policymakers, enabling persons with lived experience to review, refine, and adapt the prototype.

Participants were recruited through the Latin American National Cancer Caring Network, comprising 270 members across eight countries. Invitations to a one‐day hybrid workshop were distributed via email.

The workshop was facilitated in Spanish by lead authors and focused on four domains: (1) key support needs and content priorities, (2) delivery mechanisms, and (3) dissemination considerations (questions are provided in Supporting Information S2: Table S1). Participants engaged in small‐group discussions, either face‐to‐face or in online breakout rooms, followed by full group discussions to achieve consensus.

All discussions were audio‐recorded, transcribed verbatim in Spanish, translated into English, and analysed using inductive thematic analysis [22]. Two researchers independently coded the transcripts, with discrepancies resolved through team consensus. Findings informed the initial content and structure of the resource, which was developed into a written prototype for review in Phase 2. The study is reported in accordance with the Consolidated Criteria for Reporting Qualitative Research (COREQ) [23].

#### 2.2.2 Phase 2: Prototype Review (Santin Step 3)

To review and refine the Phase 1 prototype and gain wider stakeholder representation, ten verification workshops were conducted online or in person across Colombia. Workshops were conducted with five cancer care hospitals (*N* = 5), four universities providing oncology education (*N* = 4), and one national professional organisation (National Association of Oncology Nursing; *N* = 1).

Each workshop lasted approximately 2 hours and included a structured presentation of the Phase 1 prototype, followed by facilitated discussions exploring content, delivery and dissemination pathways. Discussions were audio‐recorded, transcribed verbatim, translated into English, and analysed thematically using the same approach employed in Phase 1. Findings informed iterative refinement of the resource.

In addition, family caregivers were invited to provide feedback through an online survey. Caregivers were recruited through a national caregiver support programme comprising 157 caregivers, all of whom received an email invitation. Closed‐ended questions assessed content clarity, relevance to caregiver needs, ease of navigation, language appropriateness, and overall value. Open‐ended questions invited participants to identify missing information, unclear or unnecessary content, and recommendations for improvement. This approach was used rather than focus groups to maximise accessibility and reduce burden for geographically dispersed caregivers, allowing them to complete the form at their own pace, rate content, and provide detailed narrative feedback. Participants received an email containing a link to a Google Form, with one reminder issued after 2 weeks. Responses were analysed thematically and incorporated into subsequent prototype refinement. Findings from Phase 2 were used to modify the Phase 1 prototype (See Table 1).

**TABLE 1:** Overview of co‐design‐informed iterative prototype development.

Iterative stage	Prototype structure	Key changes	Stakeholder input
Prototype 1 (after phase 1)	7 priority content areas	Initial content on cancer information, hospital/home care, emotional/spiritual support, alternative therapies, caregiver self‐care, myths/realities	Lead oncology nurses
Prototype 2 (after phase 2)	Expanded priority areas/10‐area resource	Added palliative and end‐of‐life care; expanded health system navigation; added legal and advocacy support, financial burden, time management, and community resources, caregiver narratives	Health care professionals, caregivers and academics
Final prototype (after phase 3)	6 integrated modules	Reorganised into a modular, non‐linear toolkit with plain language, practical guidance, multimedia, and local adaptation module	Caregivers, oncology professionals, researchers, policymakers

#### 2.2.3 Phase 3: Prototype Adaptation (Santin Step 4)

To adapt and refine the new prototype following Phase 2 review, six hybrid think‐aloud [24] workshops were conducted with stakeholders from multiple Latin American countries. Participants were recruited through cancer professional, research networks and patient organisations and included family caregivers, oncology healthcare professionals, caregiving and cancer researchers, and health policymakers. During each workshop, participants reviewed the Phase 2 prototype's structure, modules, and content while verbalising their thoughts, interpretations, comprehension difficulties, and suggestions in real time. Workshops were audio‐recorded, transcribed verbatim, translated into English where required, and analysed thematically. Findings informed further refinement of the prototype content and structure.

## 3 Results

### 3.1 Phase 1 Results: Identification of Caregiver Needs and Resource Design Priorities

Twenty‐seven oncology nurses participated in the Phase 1 co‐design workshop, representing a range of clinical roles and settings across Latin America, including hospital‐based oncology services and community care (see Supporting Information S1). Participants represented diverse geographical regions, including the central, Pacific, and Caribbean regions of Colombia, as well as Argentina and Mexico.

Analysis was organised in three overarching themes: priority content areas, preferred delivery mechanisms, and implementation considerations, which informed development of the initial prototype resource.

#### 3.1.1 Content

Participants identified seven priority content areas for inclusion within the resource (See Table 2).

- Cancer information
- Caring for patients in hospital
- Caring for patients at home
- Emotional, spiritual, and social support for caregivers
- Alternative therapies for caregivers
- Care for the caregiver
- Myths and realities about cancer

**TABLE 2:** First prototype structure developed after phase 1.

Priority content area in prototype 1	Purpose of the section	Key content included in the first prototype	Proposed formats/delivery features
Cancer information	To provide caregivers with basic, reliable and understandable information about cancer and the cancer trajectory.	General explanation of cancer; common cancer types; diagnosis and treatment pathways; cancer stages; treatment options; common questions after diagnosis.	Short educational texts, frequently asked questions, links to reliable sources.
Caring for patients in hospital	To support caregivers in understanding their role during hospital‐based cancer care.	Preparing for hospital visits; communicating with healthcare professionals; understanding hospital routines; supporting the patient during admission or treatment; questions to ask the care team.	Checklists, question prompts, practical guidance.
Caring for patients at home	To guide caregivers in managing everyday care needs in the home setting.	Symptom observation; medication routines; nutrition and hydration; hygiene and comfort; warning signs; when to contact healthcare services.	Practical tips, quick‐reference guides, downloadable materials.
Emotional, spiritual and social support	To address the emotional, relational and spiritual dimensions of cancer caregiving.	Emotional responses to cancer; family communication; social support; spirituality and meaning‐making; managing uncertainty and distress.	Caregiver narratives, reflective prompts, signposting to psychosocial and spiritual support.
Alternative and complementary therapies	To provide balanced and culturally sensitive information about complementary practices commonly used by families.	Common complementary therapies; safety considerations; communication with healthcare professionals; myths and risks related to unverified treatments.	Plain‐language explanations, FAQs, links to credible sources.
Care for the caregiver	To recognise the caregiver as a person with support needs and promote self‐care.	Caregiver burden; signs of exhaustion; self‐care strategies; asking for help; balancing caregiving with family, work and personal responsibilities.	Self‐assessment tools, wellbeing tips, caregiver testimonies.
Myths and realities about cancer	To address misinformation and culturally rooted beliefs about cancer and its treatment.	Common myths about cancer causes, treatment, contagion, pain, prognosis and complementary therapies; evidence‐informed clarification.	Myth‐versus‐reality format, short videos or infographics, simple explanatory texts.

Participants described caregiver uncertainty following diagnosis and limited access to reliable, understandable information, highlighting the need for comprehensive educational content across the cancer trajectory. While many caregiver needs were considered common across cancer types, participants also emphasised the importance of including cancer‐specific information on disease characteristics, treatment options, and symptom management.

Caregiver self‐care and psychosocial well‐being emerged as a cross‐cutting priority. Participants noted that caregivers frequently prioritised the needs of the patient at the expense of their own physical and emotional health. Consequently, supporting caregiver well‐being was viewed as equally important as providing guidance on caring for the person with cancer.

#### 3.1.2 Delivery Mechanisms

Participants strongly supported the use of digital resources to improve access to caregiver support across regions of Latin America. However, they consistently emphasised that digital resources should complement rather than replace face‐to‐face support.

A blended model of support was recommended, combining web‐based information with opportunities for direct interaction with healthcare professionals and peers. Suggested approaches included signposting caregivers to the resource during clinical consultations, educational workshops, and facilitated support groups. Group based activities were perceived as particularly valuable for sharing information about the resource, fostering emotional support, and reducing caregiver isolation.

The website can be very useful, but here not everything is solved through the internet. Many caregivers need someone to explain things to them, to look them in the eye and say: ‘You are not alone; this can be done step by step.’ Digital support helps, but it does not replace accompaniment.

Integrating peer support within the resource emerged as important, creating opportunities for caregivers to connect. Such connections were viewed as a valuable source of practical advice, emotional reassurance, and experiential knowledge.

In our countries, the family carries almost all of the care. That is why it would be very valuable for the tool not only to provide information to read, but also to help caregivers meet others, share practical tips, contacts, experiences, and feel accompanied.

Participants also highlighted the importance of using multiple communication formats within the resource to maximise accessibility and engagement. Visual and narrative approaches, including videos, short reels, and caregiver testimonials, were considered more engaging than text‐based materials and particularly beneficial for individuals with lower literacy levels.

Participants identified several factors that would influence the acceptability and implementation of a digital caregiver resource across Latin America. A key consideration was ensuring that caregivers could recognise themselves within the resource. Participants emphasised the importance of representing the diversity of Latin American caregivers, including variations in ethnicity, socioeconomic circumstances, age, gender, sexual orientation, and rural or urban residence. Real life caregiver stories and testimonials were viewed as essential for promoting inclusion.

Some caregivers do not have the time or mental space to read long texts. Between appointments, transport, home responsibilities, and medications, you need something clear, visual, and short, like a video or the story of another caregiver saying: ‘This also happened to me, and this is how I managed it’.

Participants also stressed the need for clear organisation and ease of navigation. To enhance usability, they recommended additional components, including cancer‐specific information, stages of the cancer journey, support services, frequently asked questions, updates on treatments and clinical trials, and information on complementary and alternative therapies. Participants also suggested dedicated or cross‐cutting sections for FAQs, clinical updates, clinical trials, and complementary therapies to facilitate access to information.

#### 3.1.3 Dissemination Considerations

Participants highlighted the importance of linking to credible governmental, healthcare, and community services to facilitate access to additional sources of information and support. These features were considered essential for ensuring that the resource was accessible, culturally relevant, and responsive to the diverse needs of caregivers across the region.

#### 3.1.4 Prototype Development

The findings informed development of the Phase 1 prototype, which incorporated the seven priority content areas and was organised around the cancer journey. The prototype integrated educational content, caregiver self‐management resources, links to support services, and multimedia components designed to enhance accessibility and engagement.

### 3.2 Phase 2 Results: Stakeholder Verification and Prototype Refinement

A total of 216 healthcare professionals participated in the verification workshops, representing oncology hospitals, universities, research centres, professional networks, and the National Association of Oncology Nurses (Phase 2 participant details provided in Supporting Information S1). In addition, 16 family caregivers reviewed the prototype through an online survey (Phase 2 participant details provided in Supporting Information S1). Overall, both stakeholder groups endorsed the Phase 1 prototype while identifying opportunities for further refinement.

#### 3.2.1 Content

Participants agreed that the prototype addressed many of the needs identified; however, several content gaps were identified. Healthcare professionals consistently recommended the inclusion of a dedicated module on palliative and end‐of‐life care, emphasising the importance of culturally sensitive content addressing family caregiving, spirituality, communication, and decision‐making during advanced illness.

Caregivers highlighted the need for more practical guidance on navigating fragmented healthcare systems, including accessing services, understanding insurance processes, and obtaining legal and advocacy support. They also emphasised the everyday realities of caregiving, including balancing caregiving responsibilities with employment and family life, managing financial challenges, and accessing community support resources.

A further priority was the recognition and validation of the caregiver role. Caregivers advocated for content that acknowledged them as active partners in care and incorporated lived‐experience narratives throughout the resource.

#### 3.2.2 Delivery Mechanisms

Consistent with Phase 1 findings, participants supported a blended approach to resource delivery. Caregivers expressed a strong preference for opportunities to connect with others in similar situations and recommended incorporating peer‐support mechanisms, such as moderated online groups or forums. Participants also reinforced the importance of providing information through multiple formats to accommodate differing preferences, literacy levels, and access needs. Short videos, interactive tools, embedded links, and downloadable resources were viewed as particularly valuable, while maintaining access to more traditional formats where appropriate.

#### 3.2.3 Dissemination Considerations

Both healthcare professionals and caregivers identified implementation and accessibility as critical considerations. Building on Phase 1 findings, healthcare professionals recommended integrating the resource within undergraduate and postgraduate nursing curricula and developing training strategies to support implementation and sustainability within clinical practice.

Participants also emphasised the need to ensure equitable access across diverse geographic and socioeconomic contexts. Concerns were raised that reliance on internet‐based delivery alone could exclude caregivers in underserved regions. To address this challenge, participants recommended a mixed‐format dissemination strategy incorporating web‐based resources, including downloadable and printable materials, and materials which could be disseminated via telehealth‐supported approaches.

#### 3.2.4 Prototype Refinement

Phase 2 findings informed substantive modifications to the prototype. A dedicated module on palliative and end‐of‐life care was added, while content relating to healthcare system navigation was expanded to include guidance on accessing services, understanding insurance processes, and obtaining legal and advocacy support. Additional content was developed to address the practical realities of caregiving, including time management, balancing caregiving with employment and family responsibilities, managing the financial impact of cancer care, and accessing community resources and financial assistance programmes.

Sometimes you do not know where to go, which authorization to request, or who to speak to. As caregivers, we end up learning how to navigate the system on our own, but this creates a lot of anxiety and wastes valuable time.

Family caregiver, online survey, Phase 2

It is not only about caring for the patient at home; you also have to organize appointments, authorizations, transport, money, and work responsibilities. It would be very helpful if the resource explained these issues in a simple and practical way.

Family caregiver, online survey, Phase 2

To strengthen caregiver representation, caregiver narratives, photographs, and lived‐experience testimonials were integrated throughout the resource. Delivery strategies were also refined to incorporate multiple formats, including videos, interactive content, downloadable materials, and printable resources. These refinements informed development of the revised prototype, which was subsequently reviewed during Phase 3 think‐aloud workshops.

### 3.3 Phase 3 Results: Prototype Review and Refinement

Eighty‐three participants took part in six think‐aloud workshops, including family caregivers, oncology nurses, members of chronic care networks, representatives from government and cancer research networks across Colombia (*n* = 57), Peru (*N* = 9), Argentina (*N* = 9), and Mexico (*N* = 8). Participants broadly endorsed the prototype but identified several modifications to improve usability, relevance, and implementation.

#### 3.3.1 Content Modifications

Participants recommended strengthening content related to the caregiver role, including its scope and boundaries in hospital and home settings. Additional practical guidance was requested on symptom monitoring, medication management, treatment side effects, and caregiving across the cancer trajectory. Greater attention was also recommended for the emotional and financial impacts of caregiving, including anticipatory grief from diagnosis through to end‐of‐life care. A key modification was the inclusion of module 6 which was explicitly designed to be locally adapted, providing a flexible framework that providers can populate with country‐specific guidance on accessing services, insurance processes, and support programmes within different health systems. This was viewed as necessary if the resource is to be viewed as transferable framework that can be locally adapted across different Spanish‐speaking Latin American contexts.

#### 3.3.2 Delivery Mechanisms

While participants considered the content comprehensive and credible, they consistently described the prototype as overly technical and text‐heavy. Recommendations focused on simplifying language, reducing textual density, and increasing the use of practical, action‐oriented guidance. Participants also supported greater use of multimedia formats, including videos, infographics, and downloadable resources suitable for low‐connectivity settings.

It would be better to have short videos, infographics, or downloadable checklists, because internet access is not always good and sometimes you need to review the information quickly.

Healthcare professional, think‐aloud workshop, Phase 3

#### 3.3.3 Dissemination Considerations

Participants highlighted the importance of aligning content with national clinical guidelines and ensuring robust governance processes to maintain accuracy, credibility, and cultural relevance. Particular emphasis was placed on preventing misinformation and ensuring consistency with evidence‐based practice.

#### 3.3.4 Final Prototype Refinement

Findings from the think‐aloud workshops informed the final refinement of the prototype. In response to stakeholder feedback across Phases 2 and 3, the resource evolved from a predominantly informational platform into a modular, non‐linear toolkit emphasising practical guidance, plain language, emotional support, and adaptability across diverse healthcare and community settings (See Table 3).

**TABLE 3:** Final prototype of digital resource for cancer caregivers in Latin America.

Module	Purpose	Key content	Formats and features
Module 1. Understanding the caregiver role	To support caregivers in understanding their role and expectations across the cancer trajectory.	Overview of the caregiver role Common emotional and practical challenges Changes in caregiving needs over time Recognising when to seek support	Short explanatory texts, infographics, reflection prompts
Module 2. Practical care and symptom support	To assist caregivers in managing common care needs in the home setting.	Basic symptom observation and monitoring Non‐clinical medication support guidance Recognition of warning signs When and how to contact healthcare services	Checklists, quick‐reference guides, practical tips
Module 3. Navigating health and social care systems	To empower caregivers to interact effectively with healthcare and support services.	Understanding the cancer care pathway Communicating with healthcare professionals Preparing for medical appointments Accessing available health and social resources	Step‐by‐step guides, question prompts, planning tools
Module 4. Palliative and end‐of‐life care	To provide clear and sensitive guidance during advanced illness and end‐of‐life stages.	Understanding palliative care principles Symptom comfort and dignity‐focused care Emotional support for patients and caregivers Emotional support for patients and caregivers Preparation for end‐of‐life and bereavement support	Plain‐language explanations, supportive narratives, signposting to services
Module 5. Caregiver well‐being and self‐care	To promote caregiver health and prevent emotional and physical burnout.	Recognising caregiver stress and fatigue Practical self‐care strategies Emotional coping tools When to seek professional help	Self‐assessment tools, reflective exercises, wellbeing tips
Module 6. Supportive resources and local adaptation	To connect caregivers with contextually relevant and adaptable support resources.	Community and organisational resources Guidance for local adaptation of content Printable and offline‐accessible materials	Resource lists, adaptable templates, downloadable materials

## 4 Discussion

Through an iterative co‐design process, this study developed a six‐module resource designed to address the informational and supportive needs of family caregivers of people with cancer. The final prototype encompassed understanding and managing the caregiver role, practical care and symptom support, navigating health and social care systems, palliative and end‐of‐life care, and supportive resources with local adaptation. While these modules represent distinct areas of support, participants emphasised that caregiver needs evolve across the cancer trajectory and individual circumstances. Consequently, the resource was designed to provide support that was responsive to changing needs across the disease journey and that prioritised common caregiving challenges that transcended cancer types. Consistent with previous co‐designed caregiver interventions in the United Kingdom [18], Australia [19], and Vietnam [12], stakeholders prioritised information, practical support, and healthcare navigation. However, unlike previous resources [12, 18, 19], participants strongly emphasised the importance of connecting with other caregivers and maintaining opportunities for face‐to‐face engagement health care professionals, suggesting that social connection and peer support may represent particularly important components of caregiver support within Latin American contexts. Like previous co‐design resources [12, 18, 19], this resource is needs‐based and reflects the dynamic nature of caregiving and suggests interventions may be more effective when organised around shared experiences rather than disease‐specific issues [25].

Beyond informing resource content, the co‐design process generated important insights into how support should be delivered [11, 12, 13, 14, 15, 16, 17, 18, 26]. Participants positioned digital resources as a complement to, rather than a replacement for, face‐to‐face support. This finding highlights the importance of human connection in caregiving support and suggests digital interventions should be embedded within broader care systems [27]. In areas of geographical disparities, intermittent internet access, and variable digital literacy, reliance on digital approaches may risk exacerbating existing inequities [28].

Participants highlighted the importance of user‐friendly design features, including short videos, interactive elements, embedded links, and downloadable written materials to accommodate differing needs. However, accessibility was viewed as extending beyond usability alone. Caregivers emphasised the need for resources that reflected their lived experiences through authentic imagery, testimonials, and caregiver narratives. Participants highlighted the importance of representing the diversity of Latin American caregivers across ethnicity, socioeconomic status, age, gender, sexual orientation, and place of residence. Representation was viewed as a mechanism for fostering inclusion, relatability, and emotional connection. These findings suggest digital caregiver resources should do more than provide information; they should help caregivers see themselves in the intervention [29, 30].

An important contribution of this study was the emphasis placed on implementation from the earliest stages of development. Digital health interventions frequently fail to achieve sustained adoption because implementation is considered to late. In contrast, stakeholders consistently highlighted that caregiver access to the resource would depend not only on its availability but also on its integration within existing health and social care systems. Embedding the resource within professional education, clinical guidance, and governance structures was viewed as essential for long‐term sustainability.

Although the resource was developed primarily within the Colombian context, participants identified caregiver challenges that were shared across Spanish‐speaking settings. Consequently, the resource was designed as a transferable framework rather than a country specific intervention. The inclusion of a dedicated module on supportive resources and local adaptation reflects recognition that caregiver support must balance regional relevance with responsiveness to local cultural, organisational, and healthcare contexts. The resulting resource should therefore be viewed as an adaptable model that can be modified to meet the needs of different settings while retaining a common foundation based on shared caregiver experiences.

### 4.1 Implications for Policy and Practice

The findings support involving caregivers, healthcare professionals, and stakeholders throughout development to ensure resources are relevant and implementable. They also suggest that cancer services should recognise caregivers as key recipients of support, with digital interventions embedded in broader care systems that include face‐to‐face, peer, and non‐digital options. Integrating resources into professional education, clinical guidance, and governance frameworks may help support uptake and sustainability, particularly through nursing curricula and continuing professional development.

### 4.2 Strengths and Limitations

A strength was the breadth and diversity of stakeholders across co‐design and verification phases, enhancing credibility and applicability. The iterative design allowed stakeholder input at successive stages, supporting a resource responsive to caregiver needs and health system realities. However, caregivers were not involved in the initial content‐generation workshop and were fewer than professionals in Phase 3, which may have influenced design priorities.

Further limitations should be acknowledged. Although participants were recruited from multiple Latin American countries, the majority were based in Colombia, reflecting both the location of the study and the established partnerships that facilitated recruitment. Consequently, the findings should not be interpreted as representative of the full diversity of healthcare contexts across Latin America. However, the caregiver priorities identified are broadly consistent with findings from other LMIC's [11]. Yet, contextual differences in healthcare systems and cultural expectations necessitate local adaptation, consistent with evidence from co‐design studies [11]. Male caregivers were underrepresented among caregiver participants, as support needs may differ by gender, future work should actively engage male caregivers to ensure the resource reflects their experiences. In addition, the study resulted in a written prototype rather than a fully operational digital intervention. As such, conclusions regarding usability, engagement, effectiveness, and implementation in practice cannot yet be drawn. Future research should focus on developing the digital prototype and evaluating its acceptability, effectiveness, and implementation across a wider range of settings including ownership, funding and governance arrangements.

## 5 Conclusion

Through an iterative process involving caregivers, healthcare professionals, and wider stakeholders, a six‐module resource was developed to address critical gaps in information and support. The findings highlight the importance of designing caregiver interventions that are responsive to evolving needs across the cancer trajectory, support meaningful connection and representation, and consider implementation from the outset.

## Funding

This work was supported by the Economic and Social Research Council.

## Conflicts of Interest

The authors declare no conflicts of interest.

## Supporting information


Supporting Information S1

Supporting Information S2

## Data Availability

The data that support the findings of this study are available on request from the corresponding author. The data are not publicly available due to privacy or ethical restrictions.
