# Supplementary material for: Digital Support for Cancer Caregivers in Latin America: Identifying Needs and Co‐Designing a Culturally Adapted Resource
Source: Psychooncology. 2026 Sep 17;35(9):e70609. doi: 10.1002/pon.70609 (PMC13584649; doi:10.1002/pon.70609)
Supplement: Supplementary file 1 [file PON-35-e70609-s001.docx]

Supplementary Files

**Phase 1 Step 1-2 Focus Group Questions with Cancer Nurses**

- What resource components/info is needed ?
- Whatresources are already available?
- Are they useful/beneficial for informing the development of each website component?
- What advice/support and information should be detailed in each section?
- Who should provide that information?
- How should the information be delivered? Videos? Cartoons?
- Education from expert caregivers? Narratives?
- How should we disseminate our newly developed resource?
- What are the barriers and obstacles to implementation.
- What are the research priorities for cancer carers research to inform the next steps?

**Overview of participants across the co-design phases**

| **Phase** | **Co-design activity** | **Main participant group(s)** | **Family caregivers, n** | **Healthcare professionals / stakeholders, n** | **Total, n** |
| --- | --- | --- | --- | --- | --- |
| Phase 1 | Hybrid expert co-design workshop for content generation and initial prototype development | Oncology nurses from Latin America | 0 | 27 | 27 |
| Phase 2 | Verification workshops for prototype review and refinement | Healthcare professionals from oncology hospitals, universities, research centres, professional networks and the National Association of Oncology Nursing | 0 | 216 | 216 |
| Phase 2 | Online caregiver survey for prototype review and refinement | Family caregivers recruited through *Cuidando a los Cuidadores* | 16 | 0 | 16 |
| Phase 3 | Six hybrid think-aloud workshops for further prototype review and refinement | Family caregivers, oncology nurses, chronic care network members, government and cancer research representatives, and staff from cancer care hospitals | 32 | 51 | 83 |
| **Total** |  |  | **48** | **294** | **342** |

**Demographics of Phase 1 step 1-2 Lead Oncology Nurses**

| **Variable** | **Total absolute** | **Percentage** |
| --- | --- | --- |
| Women | 26 | 96.3% |
| Men | 1 | 3.7% |
| Age |  | Mean 40.2 years; range ^30^–52 |
| Oncology specialization | 27 | 88.9% |
| Oncology experience in years |  | Mean 12.4 years; range 5–20 |

**Phase 2 Participant Characteristics**

| **Participant source / group** | **Engagement activity** | **Main profile** | **Count** | **Percentage** |
| --- | --- | --- | --- | --- |
| Healthcare professionals / stakeholders | Verification workshops for prototype review and refinement | Oncology hospitals, universities, research centres, professional networks and oncology nursing organisation | 216/232 | 93.1% |
| Family caregivers | Online survey for prototype review and refinement | Family caregivers recruited through Cuidando a los Cuidadores | 16/232 | 6.9% |
| **Total Phase 2 participants** |  |  | **232/232** | **100.0%** |

**Characteristics of healthcare professionals and stakeholders participating in Phase 2 verification workshops (N = 216)**

| Characteristic | n (%) or Mean (Range) |
| --- | --- |
| Demographic characteristics | |
| Women | 190 (88.0) |
| Men | 26 (12.0) |
| Age (years) | 41.6 (29–61) |
| Professional experience in oncology/cancer care (years) | 11.8 (3–26) |
| Highest qualification | |
| Professional degree / no postgraduate training | 4 (1.9) |
| Postgraduate specialization in oncology or related area | 129 (59.7) |
| Master's degree | 43 (19.9) |
| Doctoral degree or doctoral training | 17 (7.9) |
| Medical specialty in oncology or related field | 23 (10.6) |
| Main professional role | |
| Oncology nursing service coordinators | 48 (22.2) |
| Oncology nurse navigators | 42 (19.4) |
| Direct care nurses in chemotherapy, oncology or inpatient services | 39 (18.1) |
| Nursing leadership and patient education nurses | 18 (8.3) |
| Oncologists and physicians | 23 (10.6) |
| Researchers and research coordinators | 24 (11.1) |
| Epidemiologists and methodologists | 9 (4.2) |
| Other clinical, academic or administrative stakeholders | 13 (6.0) |
| Main practice/work setting | |
| Chemotherapy and ambulatory oncology services | 72 (33.3) |
| Inpatient oncology and haemato-oncology services | 44 (20.4) |
| Patient navigation and patient/caregiver education | 43 (19.9) |
| Research, epidemiology and academic networks | 41 (19.0) |
| Nursing leadership, service coordination or professional organisations | 16 (7.4) |

**Family caregiver profile, online survey, n = 16**

| **Variable** | **Count** | **Percentage / descriptive** |
| --- | --- | --- |
| Total family caregivers | 16 | 100.0% |
| Women | 15/16 | 93.8% |
| Men | 1/16 | 6.3% |
| Age | 16 | Mean 41.3 years; range 25–62 |
| Elementary education | 2/16 | 12.5% |
| High school education | 7/16 | 43.8% |
| University education | 7/16 | 43.8% |
| Housewife | 7/16 | 43.8% |
| Informal job | 6/16 | 37.5% |
| Unemployed | 1/16 | 6.3% |
| Retired | 1/16 | 6.3% |
| Self-employed | 1/16 | 6.3% |
| Daily hours of care | 16 | Mean 9.6 hours/day; range 8–11 |
| Time as caregiver | 16 | Mean 11.6 months; range 9–14 |

**Patient cancer diagnosis reported by family caregivers Phase 2, n = 16**

| **Cancer diagnosis** | **Count** | **Percentage** |
| --- | --- | --- |
| Breast cancer | 3/16 | 18.8% |
| Gastric cancer | 2/16 | 12.5% |
| Haematological cancer | 2/16 | 12.5% |
| Cervical cancer | 2/16 | 12.5% |
| Prostate cancer | 2/16 | 12.5% |
| Lung cancer | 2/16 | 12.5% |
| Colorectal cancer | 2/16 | 12.5% |
| Skin cancer / melanoma | 1/16 | 6.3% |

**Phase 3 participant characteristics**

**Participant source and engagement activity Phase 3**

| **Participant source / group** | **Engagement activity** | **Main profile** | **Count** | **Percentage** |
| --- | --- | --- | --- | --- |
| Family caregivers | Six hybrid think-aloud workshops for prototype review and refinement | Family caregivers of people with cancer from Latin America | 32/83 | 38.6% |
| Healthcare professionals / stakeholders | Six hybrid think-aloud workshops for prototype review and refinement | Oncology nurses, chronic care network members, government and cancer research representatives, and staff from cancer care hospitals | 51/83 | 61.4% |
| **Total Phase 3 participants** |  |  | **83/83** | **100.0%** |

**Family caregiver profile phase 3, n = 32**

| **Variable** | **Count** | **Percentage / descriptive** |
| --- | --- | --- |
| Total family caregivers | 32 | 100.0% |
| Women | 28/32 | 87.5% |
| Men | 4/32 | 12.5% |
| Age | 32 | Mean 43.8 years; range 24–68 |
| Elementary education | 5/32 | 15.6% |
| High school education | 14/32 | 43.8% |
| Technical or vocational education | 5/32 | 15.6% |
| University education | 8/32 | 25.0% |
| Employed or self-employed | 11/32 | 34.4% |
| Informal work | 7/32 | 21.9% |
| Housewife / unpaid household work | 10/32 | 31.3% |
| Unemployed | 2/32 | 6.3% |
| Retired | 2/32 | 6.3% |
| Daily hours of care | 32 | Mean 9.1 hours/day; range 4–16 |
| Time as caregiver | 32 | Mean 18.4 months; range 3–60 |
| Urban residence | 24/32 | 75.0% |
| Rural or peri-urban residence | 8/32 | 25.0% |

**Caregiver relationship to the person with cancer phase 3, n = 32**

| **Relationship** | **Count** | **Percentage** |
| --- | --- | --- |
| Daughter or son | 13/32 | 40.6% |
| Spouse or partner | 9/32 | 28.1% |
| Parent | 4/32 | 12.5% |
| Sibling | 3/32 | 9.4% |
| Other relative | 3/32 | 9.4% |

**Patient cancer diagnosis reported by family caregivers phase 3, n = 32**

| **Cancer diagnosis** | **Count** | **Percentage** |
| --- | --- | --- |
| Breast cancer | 6/32 | 18.8% |
| Haematological cancer | 5/32 | 15.6% |
| Gastric cancer | 4/32 | 12.5% |
| Lung cancer | 4/32 | 12.5% |
| Colorectal cancer | 4/32 | 12.5% |
| Cervical cancer | 3/32 | 9.4% |
| Prostate cancer | 3/32 | 9.4% |
| Other cancer types | 3/32 | 9.4% |

**Healthcare professional and stakeholder profile phase 3, n = 51**

| **Variable** | **Count** | **Percentage / descriptive** |
| --- | --- | --- |
| Total healthcare professionals / stakeholders | 51 | 100.0% |
| Women | 43/51 | 84.3% |
| Men | 8/51 | 15.7% |
| Age | 51 | Mean 42.9 years; range 30–62 |
| Professional experience in oncology, caregiving, research or policy | 51 | Mean 13.2 years; range 4–28 |
| Postgraduate specialization in oncology, palliative care, public health or related area | 25/51 | 49.0% |
| Master’s degree | 14/51 | 27.5% |
| Doctoral degree or doctoral training | 7/51 | 13.7% |
| Medical specialty in oncology or related field | 5/51 | 9.8% |

**Stakeholder group Phase 3 ( HCP), n = 51**

| **Stakeholder group** | **Count** | **Percentage** |
| --- | --- | --- |
| Oncology nurses | 16/51 | 31.4% |
| Cancer care hospital staff | 10/51 | 19.6% |
| Chronic care network members | 8/51 | 15.7% |
| Cancer and caregiving researchers | 7/51 | 13.7% |
| Government / policy or decision-making representatives | 6/51 | 11.8% |
| Patient or caregiver organisation representatives | 4/51 | 7.8% |

**Main professional role Phase 3(HCP), n = 51**

| **Main professional role** | **Count** | **Percentage** |
| --- | --- | --- |
| Oncology nurse navigator | 10/51 | 19.6% |
| Oncology nursing service coordinator | 8/51 | 15.7% |
| Direct care nurse in oncology or haemato-oncology services | 6/51 | 11.8% |
| Patient education or caregiver education professional | 5/51 | 9.8% |
| Oncologist or physician | 5/51 | 9.8% |
| Researcher or research coordinator | 7/51 | 13.7% |
| Public health, policy or decision-making representative | 6/51 | 11.8% |
| Patient/caregiver advocacy representative | 4/51 | 7.8% |

**Country represented Phase 3, n = 83**

| **Country** | **Count** | **Percentage** |
| --- | --- | --- |
| Colombia | 57/83 | 68.7% |
| Peru | 9/83 | 10.8% |
| Argentina | 9/83 | 10.8% |
| Mexico | 8/83 | 9.6% |
