# Supplementary material for: Digital Support for Cancer Caregivers in Latin America: Identifying Needs and Co‐Designing a Culturally Adapted Resource
Source: Psychooncology. 2026 Sep 17;35(9):e70609. doi: 10.1002/pon.70609 (PMC13584649; doi:10.1002/pon.70609)
Supplement: Supplementary file 2 [file PON-35-e70609-s002.docx]

**Supplementary Materials**

**Supplementary Table 1. Phase 1 Steps 1–2 focus group questions with cancer nurses**

| **No.** | **Question** |
| --- | --- |
| 1 | What resource components/information are needed? |
| 2 | What resources are already available? |
| 3 | Are they useful/beneficial for informing the development of each website component? |
| 4 | What advice/support and information should be detailed in each section? |
| 5 | Who should provide that information? |
| 6 | How should the information be delivered (e.g., videos, cartoons)? |
| 7 | Should education from expert caregivers and/or narratives be included? |
| 8 | How should the newly developed resource be disseminated? |
| 9 | What are the barriers and obstacles to implementation? |
| 10 | What are the research priorities for cancer carers research to inform the next steps? |

**Supplementary Table 2. Co-design activities and participants across study phases**

| **Phase** | **Co-design activity** | **Participant group** | **n** | **% within phase** |
| --- | --- | --- | --- | --- |
| Phase 1 | Hybrid expert co-design workshop for content generation and initial prototype development | Oncology nurses from Latin America | 27 | 100.0 |
| Phase 2 | Verification workshops for prototype review and refinement | Healthcare professionals/stakeholders from oncology hospitals, universities, research centres, professional networks and the national oncology nursing organisation | 216 | 93.1 |
| Phase 2 | Online caregiver survey for prototype review and refinement | Family caregivers recruited through Cuidando a los Cuidadores | 16 | 6.9 |
| Phase 3 | Six hybrid think-aloud workshops for prototype review and refinement | Family caregivers | 32 | 38.6 |
| Phase 3 | Six hybrid think-aloud workshops for prototype review and refinement | Oncology nurses, chronic care network members, government/policy and cancer research representatives, and cancer care hospital staff | 51 | 61.4 |

*Note.* Across all phases, 342 stakeholders participated: 48 family caregivers and 294 healthcare professionals/stakeholders. Phase 2 included 232 participants (216 healthcare professionals/stakeholders and 16 caregivers). Phase 3 included 83 participants (32 caregivers and 51 healthcare professionals/stakeholders). Phase 3 participants represented Colombia (57/83, 68.7%), Peru (9/83, 10.8%), Argentina (9/83, 10.8%), and Mexico (8/83, 9.6%).

**Supplementary Table 3. Characteristics of healthcare professionals and stakeholders across co-design phases**

| **Characteristic** | **Phase 1 Oncology nurses (n = 27)** | **Phase 2 Healthcare professionals/stakeholders (n = 216)** | **Phase 3 Healthcare professionals/stakeholders (n = 51)** |
| --- | --- | --- | --- |
| Women, n (%) | 26 (96.3) | 190 (88.0) | 43 (84.3) |
| Men, n (%) | 1 (3.7) | 26 (12.0) | 8 (15.7) |
| Age, mean (range), years | 40.2 (30–52) | 41.6 (29–61) | 42.9 (30–62) |
| Relevant professional experience, mean (range), years | 12.4 (5–20) in oncology | 11.8 (3–26) in oncology/cancer care | 13.2 (4–28) in oncology, caregiving, research or policy |
| Highest qualification / postgraduate preparation | Not reported | Professional degree/no postgraduate training: 4 (1.9%); postgraduate specialization in oncology or related area: 129 (59.7%); master's degree: 43 (19.9%); doctoral degree/training: 17 (7.9%); medical specialty in oncology or related field: 23 (10.6%) | Postgraduate specialization in oncology, palliative care, public health or related area: 25 (49.0%); master's degree: 14 (27.5%); doctoral degree/training: 7 (13.7%); medical specialty in oncology or related field: 5 (9.8%) |
| Main professional roles | Lead oncology nurses | Oncology nursing service coordinators: 48 (22.2%); oncology nurse navigators: 42 (19.4%); direct care nurses: 39 (18.1%); nursing leadership/patient education nurses: 18 (8.3%); oncologists/physicians: 23 (10.6%); researchers/research coordinators: 24 (11.1%); epidemiologists/methodologists: 9 (4.2%); other clinical, academic or administrative stakeholders: 13 (6.0%) | Oncology nurse navigator: 10 (19.6%); oncology nursing service coordinator: 8 (15.7%); direct care nurse: 6 (11.8%); patient/caregiver education professional: 5 (9.8%); oncologist/physician: 5 (9.8%); researcher/research coordinator: 7 (13.7%); public health/policy representative: 6 (11.8%); patient/caregiver advocacy representative: 4 (7.8%) |
| Main work setting / stakeholder group | Not reported | Chemotherapy/ambulatory oncology: 72 (33.3%); inpatient oncology/haemato-oncology: 44 (20.4%); patient navigation/patient-caregiver education: 43 (19.9%); research/epidemiology/academic networks: 41 (19.0%); nursing leadership/service coordination/professional organisations: 16 (7.4%) | Oncology nurses: 16 (31.4%); cancer care hospital staff: 10 (19.6%); chronic care network members: 8 (15.7%); cancer/caregiving researchers: 7 (13.7%); government/policy representatives: 6 (11.8%); patient/caregiver organisation representatives: 4 (7.8%) |

*Note.* Percentages are based on the phase-specific denominator.

**Supplementary Table 4. Characteristics of family caregivers participating in Phases 2 and 3**

| **Characteristic** | **Phase 2 online survey (n = 16)** | **Phase 3 think-aloud workshops (n = 32)** |
| --- | --- | --- |
| Women, n (%) | 15 (93.8) | 28 (87.5) |
| Men, n (%) | 1 (6.3) | 4 (12.5) |
| Age, mean (range), years | 41.3 (25–62) | 43.8 (24–68) |
| Education, n (%) | Elementary: 2 (12.5%); high school: 7 (43.8%); university: 7 (43.8%) | Elementary: 5 (15.6%); high school: 14 (43.8%); technical/vocational: 5 (15.6%); university: 8 (25.0%) |
| Employment/occupation, n (%) | Housewife: 7 (43.8%); informal job: 6 (37.5%); unemployed: 1 (6.3%); retired: 1 (6.3%); self-employed: 1 (6.3%) | Employed/self-employed: 11 (34.4%); informal work: 7 (21.9%); housewife/unpaid household work: 10 (31.3%); unemployed: 2 (6.3%); retired: 2 (6.3%) |
| Daily hours of care, mean (range) | 9.6 (8–11) | 9.1 (4–16) |
| Time as caregiver, mean (range), months | 11.6 (9–14) | 18.4 (3–60) |
| Residence, n (%) | Not reported | Urban: 24 (75.0%); rural/peri-urban: 8 (25.0%) |
| Relationship to person with cancer, n (%) | Not reported | Daughter/son: 13 (40.6%); spouse/partner: 9 (28.1%); parent: 4 (12.5%); sibling: 3 (9.4%); other relative: 3 (9.4%) |
| Patient cancer diagnosis, n (%) | Breast: 3 (18.8%); gastric: 2 (12.5%); haematological: 2 (12.5%); cervical: 2 (12.5%); prostate: 2 (12.5%); lung: 2 (12.5%); colorectal: 2 (12.5%); skin cancer/melanoma: 1 (6.3%) | Breast: 6 (18.8%); haematological: 5 (15.6%); gastric: 4 (12.5%); lung: 4 (12.5%); colorectal: 4 (12.5%); cervical: 3 (9.4%); prostate: 3 (9.4%); other cancer types: 3 (9.4%) |

*Note.* Percentages are based on the phase-specific caregiver denominator.
